# Easier Said Than Done: The Challenge to Teach “Personal Recovery” to Mental Health Professionals Through a Short, Targeted and Structured Training Programme

**DOI:** 10.1007/s10597-021-00910-w

**Published:** 2021-11-08

**Authors:** Laura Giusti, Donatella Ussorio, Anna Salza, Massimo Casacchia, Rita Roncone

**Affiliations:** Department of Life, Health and Environmental Sciences, University Unit Rehabilitation Treatment, Early Interventions in Mental Health, Hospital S. Salvatore, University of L’Aquila, Building Delta 6, Coppito, 67100 L’Aquila, Italy

**Keywords:** Personal recovery, Training, Mental health professionals, Mental illness, Consumers

## Abstract

This study assesses the effectiveness of our short Personal Recovery Training Program (PRTP) for mental health professionals. Fifty-two healthcare professionals from Italian mental health services and forty students in psychiatric rehabilitation completed the Recovery Knowledge Inventory (RKI) pre- and post-training, divided into two groups: the PRTP (N = 45) and the Family Psychoeducational Training Program (FPTP; N = 47). Participants’ understanding of personal recovery improved more significantly for those in the PRTP than for those in the FPTP group in two domains, “Roles and responsibilities” and “Non-linearity of the recovery process”; the FPTP group showed a significant improvement in the “Role of self-definition and peers in recovery” domain. Two consumers were involved in the PRTP and represented a resource to help participants understand the personal recovery process. Our findings indicate that a brief PRTP supported by consumers can improve staff and students' recovery orientation. The translation of the training into clinical practice remains unevaluated.

## Introduction

From the perspective of the person with mental illness, recovery means gaining and retaining hope, understanding one’s abilities and disabilities, engagement in active life, personal autonomy, social identity, meaning and purpose in life, and a positive sense of self (WHO, 2012). Importantly, recovery is defined by the person themself and not other people’s definition of what recovery means (Patel et al., 2018; Slade & Longden, 2015a).

As a key term to define the scope of mental health by the Lancet Commission, this recent definition of “personal recovery” (Patel et al., 2018) seems to make it difficult to scientifically investigate this important construct that has garnered considerable attention in the two last decades.

The first definition of personal recovery (Anthony, 1993) defined it as “a way of living a satisfying, hopeful, and contributing life even in the presence of limitations caused by illness”. Thus, in contrast to clinical or social recovery, comprising a reduction or absence of symptoms and a significant improvement in occupational and social functioning, personal recovery was defined as a process that individuals go through to live a satisfying life and achieve life goals (Lemos-Giraldez et al., 2015), a process of helping people to live a life ‘beyond illness’—i.e. to recover a meaningful life, with or without symptoms is the traditional meaning applied to ‘personal’ recovery (Slade, 2009).

The difficulty in establishing a more reliable definition of the construct and its assessment could explain the sometimes contradictory research results on the role and predictive value of the personal recovery on the history of illness. Several studies, which have examined personal recovery among subjective and personal resources, observed that personal recovery positively mediates the impact of symptoms and cognitive impairment on real-life functioning in subjects with schizophrenia (Galderisi et al., 2014, 2016; Rossi et al., 2017, 2018). On the other side, lower cognitive and clinical insight, lower social functioning, and total independence from the illness condition and the functional status were reported as significant predictors of personal recovery (Chan et al., 2018; Giusti et al., 2015; Roe et al., 2011).

Liberman and Kopelowicz’s (Liberman & Kopelowicz, 2005) consideration of the concept of recovery being still “in search of research” sounds realistic; however, if it seems difficult to share a common conceptual “personal recovery” paradigm (Cleary & Dowling, 2009), it seems even more challenging to “teach” this vision of recovery orientation, which require a whole-systems approach, to healthcare professionals (Le Boutillier et al., 2015; Leamy et al., 2011).

The challenge to improve the attitude of mental health professionals towards adopting a recovery orientation model was faced by several Authors. Using a self-report measure, Crowe et al. (Crowe et al., 2006) examined the impact of a two-day, recovery-based training program for mental health workers on the knowledge, attitudes, and hopefulness related to the recovery prospects of people with enduring mental illness. The results showed a good improvement in attitudes, hopefulness, and knowledge regarding principles of recovery and belief in the effectiveness of collaboration and consumer autonomy support, motivation enhancement, needs assessment, goal striving, and homework use. Some Authors (Meehan & Glover, 2009; Stratford et al., 2012) developed and trialled specific training for personal recovery and wellbeing and stressed the importance of a consumer-led recovery training program, demonstrating significant gains in knowledge of recovery-based practice. Feenay et al. (Feeney et al., 2013) evaluated a recovery-focused teaching program for medical students in psychiatry, evaluating them before and after either a six-week traditional placement (exposure to acutely unwell patients in inpatient settings and attendance at outpatient follow-up appointments where symptoms and medication compliance were the focus) or recovery-focused clinical placement in psychiatry. Following the recovery-focused clinical placement, the medical students’ recovery knowledge significantly increased and they showed more positive attitudes toward mental illness and psychiatry, greater optimism, and more holistic concepts of recovery from mental illness compared with students who underwent a traditional placement. Slade et al. (Slade et al., 2014) identified 10 empirically-validated interventions which support recovery by targeting five key recovery processes: connectedness, hope, identity, meaning and empowerment (the CHIME framework) including, for example, peer support workers, advance directives, wellness recovery action planning, illness management and recovery, recovery education programs and supported housing. A pro-recovery manualised intervention called the REFOCUS intervention—for use in mental health services—was developed (Slade & Longden, 2015b; Slade et al., 2015a, b), including two components: recovery promoting relationships and working practices. The REFOCUS intervention was successfully employed in training community-based adult mental health teams, showing as primary outcome their support in the recovery of users with psychosis (Macpherson et al., 2015; Slade et al., 2011, 2015a, b; Wallace et al., 2016). In Australia, an adapted version of the original training, the REFOCUS-PULSAR training, was conducted in community mental health services, showing that the training conducted with 190 staff had a small but significant effect on the recovery of a large sample of users and promotion of recovery-oriented practice (Meadows et al., 2019). The same training, REFOCUS-PULSAR, was used to improve personal recovery outcomes in adults with mental health problems consulting Australian general practitioners, GPs (Enticott et al., 2021). GPs underwent an 8-h training, with the availability of support sessions for 1 year, in recovery-oriented practice, trainers including a psychiatrist, a person with lived experience of mental health problems and training experience, and, less frequently, an experienced family/carer worker. Results were encouraging displaying better clients outcomes followed by introducing GPs to recovery-oriented practice in routine practice conditions.

As in Western health settings (Hungerford et al., 2015), mental health professionals in Italy are familiar with the terms “recovery” and “personal recovery” which they adopted tout-court into their language. Daily, they try to translate it into their practice as a user-centered approach, framed by the principles of self-determination and collaboration and underpinned by the notion of hope and optimism.

A recent Italian investigation involving 426 mental healthcare professionals and students of psychiatric rehabilitation techniques showed a good global orientation toward recovery, reflecting a recovery-oriented biopsychosocial perspective in their attitudes (Giusti et al., 2019). The study included students of “psychiatric rehabilitation techniques”, who were undergoing a three-year academic curriculum to be skilled in properly administering psychosocial interventions. These technicians represent an innovative professional workforce in mental healthcare that has not yet established outside Italy, and a specific mental-health academic and professional profile, created after the passage of Law 180 (Pingani et al., 2013; Roncone et al., 2016), to work in a psychiatric community team. The study reported that these students and younger mental health workers seemed to show a higher cognitive openness and flexibility than the more experienced colleagues, who still had some difficulty accepting the recovery dimension of “non-linearity” and their users’ well-being “beyond” treatment adherence and psychopathological stability (Giusti et al., 2019).

In this study we adopted the personal recovery model according to Bedregal et al. (2006), a model already tested in Giusti et al. study (Giusti et al., 2019), referred to the issues related to the provision of clinical and rehabilitative services oriented to promoting recovery: consumer directedness, individual nature of recovery, cultural competence, self-determination, strengths-based care, choice and risk-taking, illness and symptom management, incorporation of illness into the sense of self, involvement in meaningful activities, overcoming stigma, redefining self, hope, and the non-linear nature of the recovery process”. Based on the aforementioned model, we developed our short and targeted Personal Recovery Training Program (PRTP) for mental health professionals to improve the understanding of the users’ “personal recovery” paradigm, in a country such as Italy that has practiced community mental healthcare for 40 years.

The aim of our study was to preliminary examine the effectiveness of the PRTP for mental health professionals (psychiatrists, psychologists, nurses, and psychiatric rehabilitation technicians) and students of psychiatric rehabilitation techniques compared to the Family Psychoeducational Training Program (FPTP), which was used as comparison program. We hypothesized that exposure to a specific training program on personal recovery would significantly improve attitudes towards recovery-oriented practices more than a broad-based training course. We were also interested in verifying whether greater improvements would be seen among the students’ and younger professionals’ attitudes regarding personal recovery than among the older mental health professionals.

## Methods

### Study Participants and Procedure

In total, 52 mental health professionals from Italian mental health services and 40 students in psychiatric rehabilitation techniques completed the Recovery Knowledge Inventory (RKI) (Bedregal et al., 2006) at the beginning and end of two different training programs. Twenty-five mental health professionals and 20 students attended the structured PRTP (experimental group), lasting one day for eight hours, and 27 mental health professionals and 20 students participated in a structured training course on family integrated psychoeducational treatments lasting six days (eight hours per day) (FPTP, as Comparison Group). Both courses included the voluntary participation of mental health workers from every part of our country: courses registration included the expression of interest for the training event and the sending of a short curriculum vitae. Any of the trainees in the PRTP asked to train in the FPTP intervention also. The participants’ main socio-demographic data (gender, age) and information regarding professional role, level of experience (years), work setting and previous exposure to recovery training were recorded. Informed consent was obtained from all study participants.

### Personal Recovery Training Program, PRTP

The training, which was organized on September 7, 2015 and directed by the L’Aquila Psychiatric University Unit of the Department of Life, Health and Environmental Sciences*,* was conducted by leading mental health university and National Health System (NHS) experts. Two consumers were involved as teachers and tutors. Both the consumers were part of the board of the Italian section of the World Association for Psychosocial Rehabilitation and were experienced in teaching, tutoring and talking about their experiences. Table 1 describes the PRTP course, proposed during a national meeting entitled “The recovery process: A new paradigm for mental health services. Easier said than done”.

**Table 1:** Description of the Personal Recovery Training Program, PRTP

Time (h)	Teaching method	Aim	Activities
½ hour	Lecture	To illustrate the evolution of the concept of outcome in mental health	- Didactic presentation
1 h	Small groups work assisted by a tutor	To improve knowledge and practice of tools to measure the degree of adhesion of the mental health professionals to principles of personal recovery and their effective application in daily practice	- Self-assessment of the knowledge and attitudes about personal recovery from mental illness of participants by the RKI (Bedregal et al., 2006) - Reading of “Ten top tips’ for recovery-oriented practice” (Shepherd et al., 2008) - Self-assessment of participants skills by the “Ten top tips’ for recovery-oriented practice” - Discussion
½ hour	Lecture	To share the basic concepts and principles of the “personal recovery” process	- Didactic presentation
½ hour	Lecture	To illustrate the influence of the mental health professionals’ work on the users’ recovery process	- Didactic presentation
1 h	Small groups work assisted by a tutor	To improve knowledge and practice of tools to measure and monitor the recovery style and recovery process of users, “Recovery is best judged by the person living with the experience” (Slade & Longden, 2015a, b)	- Instrument for assessing and monitoring of personal recovery from mental illness and their scoring - Discussion
1 h	Users personal reports	To experience the support and the difficulties appreciated by the users in their recovery process	- Personal reports on their recovery process by the two consumers involved in the program
½ hour	Lecture	To illustrate the impact of personal, psychosocial and mental healthcare variables on the user’s recovery process	- Didactic presentation
½ hour	Lecture	To illustrate the evidence-based integrated treatments and healthy lifestyles for subjects affected by mental disorders To illustrate the need to support people suffering from mental disorders, especially young people with psychosis, during their recovery process, through programs aimed at improving their mental and physical health	- Didactic presentation
1 ½ hour	Small groups work assisted by a tutor	To empower teams to translate recovery ideas into practice and to utilize the skills and resources, both those providing and those using services, to develop innovative ways of promoting recovery and recovery environments	- Reading of “The team recovery implementation plan: A framework for creating recovery-focused services” (Repper & Perkins, 2013) - Discussion and agreement on criteria suggested by the plan - Self-assessment of the degree of implementation of such criteria in their services
½ hour	Large group	To identify the main quality requirements and daily practices of a recovery-oriented mental health service and develop a large consensus about the main criteria	- Discussion - Formulation of a list of five main criteria characterising recovery-focused services
½ hour	Lecture	To promote adherence to the model of recovery to determine radical changes in the organisation of mental health services and the mental health professionals	- Didactic presentation

### Family Psychoeducational Training Program, FPTP

The FPTP, which included a structured 6-day training course by Falloon (Falloon, 1994) on family cognitive-behavioural psychoeducational treatment was organised at L’Aquila (Italy) on 14–19 September 2015. The course was conducted by one of the Author R.R. and the training method is described in Falloon et al. (Falloon et al., 1999). The approach included the following strategies: individual evaluation of each member of the family, assessment of the communication skills and problem-solving capacity of the family as a whole, personal and family objective setting, education regarding the nature of the disorder and its biomedical and psychosocial treatment, identification of early warning signs, improvement of communication skills, structured problem solving and social skills training.

### Instruments

The training outcome was assessed using the Recovery Knowledge Inventory, RKI (Bedregal et al., 2006), in its Italian version (Basso et al., 2016). The RKI is a quick and easy to administer instrument, consistent with the conceptual paradigm of personal recovery and well-known in scientific literature. The scale comprises 20 statements measured on a five-point Likert scale and assesses four different domains of understanding for recovery in mental health: (a) “Roles and responsibilities in recovery” (7 items; range score: 7–35), regarding risk-taking, decision-making and the various and respective roles and responsibilities of people in recovery and behavioural health providers (e.g. people with mental illness should take responsibilities of everyday life); (b) “Non-linearity of the recovery process” (6 items; range score: 6–30), regarding the role of illness and symptom management and the non-linear nature of recovery (e.g. recovery is characterised by a person making gradual steps forward with major steps backward); (c) “Roles of self-definition and peers in recovery” (5 items; range score: 5–25), regarding a person’s activities in defining an identity for him/herself, and a life that goes beyond that of “mental patient”, including the valuable roles that peers can play in this process (e.g. the pursuit of hobbies and leisure activities is important for recovery); and (d) “Expectations regarding recovery” (2 items; range score: 2–10), regarding expectations (e.g. everyone is capable of actively participating in the recovery process). Each item is rated on a five-point scale ranging from 1 (strongly disagree) to 5 (strongly agree). Higher scores represent greater orientation to the concept of recovery (cut-off scores are not reported in the literature). Cronbach’s alpha relating to each of the subscales is reported by the authors (Bedregal et al., 2006) as follows: “Roles and responsibilities in recovery” (0.81), “Non linearity of the recovery process” (− 0.70), “Role of self-determination and peers in recovery” (0.63) and “Expectations regarding recovery” (− 0.47).

### Data Analysis

Chi-squared test and one-way analyses of variance (ANOVA) were conducted to examine differences among socio-demographic variables. Five 2 × 2 factorial ANOVAs were performed to compare the pre-test scores (subscale and total) for the RKI with the scores obtained immediately post training. These enabled us to assess the main effects for time (pre vs. post) and group (PRTP vs. Psychoeducational Training), as well as the interaction between group and time. Participants’ age was included in the model as a covariate (as a variable strictly connected to the level of experience in the mental health field). The estimated effect size (η^2^p) was also calculated, and a level of significance of *p* < 0.05 was adopted. Statistical analyses were performed using SPSS 26.0 (SPSS Inc., Chicago, IL, USA).

## Results

Table 2 describes the main socio-demographic and professional data of the participants.

**Table 2:** Socio-demographic characteristics of the study participants divided into groups

	Personal Recovery Training Program (N = 45)	Family psychoeducational training program (N = 47)
*Gender, n (%)*		
Men	10 (22.2)	7 (15)
Women	35 (77.8)	40 (85)
Age, years—mean (SD)	30 (9.34)	32.1 (12.58)
*Work setting, N (%)*		
Acute unit (admission wards in inpatient psychiatric facilities)	13 (28.9)	5 (10.6)
Community (community mental health teams)	12 (26.6)	22 (46.8)
University traineeship in psychiatric units	20 (44.4)	20 (42.6)
*Professions, N (%)*		
Psychiatrists	11 (24.4)	4 (8.5)
Nurses	3 (6.7)	5 (10.6)
Psychologists	2 (4.4)	4 (8.5)
Psychiatric rehabilitation technicians	9 (20)	14 (29.8)
Students of psychiatric rehabilitation techniques	20 (44.4)	20 (42.6)
*Years worked in mental health, n (%) (students excluded)*		
< 15 years	22 (88)	18 (67)
> 15 years	3 (12)	9 (33)

Our total sample comprised university students of psychiatric rehabilitation techniques (more than 40% in both groups) and young mental health professionals (only 20% older than 40 years), more than 80% women, with a relatively short-term working experience, and without statistically significant differences between the participants in the two groups. All participants reported that they had not received previous “formal training” in personal recovery principles, and those with exposure to this concept gained their knowledge through informal methods rather than structured programs.

Differences in the overall KI scale scores produced a significant time x group interaction. Improvements of the personal recovery concept, as measured by the RKI total score, increased significantly more among those undertaking the PRTP than among those in the FPTP group [F(1,90), = 7.39; *p* < 0.001] (Table 3). Significant interactions were found in two domains of the RKI, with greater improvement evident for the PRTP group than for the FPTP: “Roles and Responsibilities” [F(1,90), = 10.20; *p* < 0.002]; “Non-linearity of the recovery process” [F(1,90), = 12.23; *p* < 0.001].

**Table 3:** Means and standard deviations Pre-Training (T0) and Post-Training (T1) for the experimental, PRTP, and the comparison groups, FPTP

RECOVERY KNOWLEDGE—DOMAINS (mean, SD)	Personal Recovery Training Program (n = 45)		Family psychoeducational training program (n = 47)		F value	p	ƞp^2^	F value	p	ƞp^2^^a^
	T0	T1	T0	T1						
Roles and responsibilities	3.39 (0.46)	3.85 (0.37)	3.38(0.51)	3.41 (0.69)	Time 12.70** Group 4.74* Interaction 10.20**	0.001 0.032 0.002	0.118	Time 4.84* Group 3.54 Interaction 8.89**	0.031 0.064 0.004	0.106
Non-linearity of the recovery process (range 6–30)	2.41 (0.50)	2.81 (0.72)	2.25(0.43)	2.17 (0.52)	Time 4.94* Group 14.70** Interaction 12.23**	0.029 0.000 0.001	0.139	Time 0.992 Group 14.96** Interaction 11.48**	0.322 0.000 0.001	0.133
Role of self-definition and peers in recovery (range 5–25)	4.33 (0.35)	4.40 (0.50)	4.06 (0.63)	4.46 (0.51)	Time 16.78** Group 1.106 Interaction 8.56**	0.000 0.296 0.005	0.101	Time 0.006 Group 0.673 Interaction 7.048*	0.937 0.415 0.010	0.086
Expectations regarding recovery (range 2–10)	3.29 (0.76)	3.95 (0.72)	2.96 (0.92)	3.30 (0.93)	Time 20.69** Group 9.30** Interaction 2.25	0.000 0.003 0.137	0.000	Time 0.469 Group 7.53** Interaction 3.701	0.496 0.008 0.058	0.046
Total RKI (range 20–100)	3.35 (0.35)	3.74 (0.34)	3.16 (0.41)	3.33 (0.37)	Time 47.015** Group 15.071** Interaction 7.39**	0.000 0.000 0.008	0.119	Time 1.491 Group 21.39** Interaction 9.73**	0.226 0.000 0.003	0.115

*p < .05

**p < .01

^a^Controlling for age variable

Compared to the PRTP, the FPTP showed a better improvement in the domain of “Role of self-definition and peers in recovery” [F(1,90), = 8.56; *p* < 0.005].

No significant interaction time × group was found on RKI total score and component “Expectations regarding recovery”. No statistically significant differences were found when age was included as a covariate in the model.

## Discussion

Knowledge and attitudes about the personal recovery paradigm, as identified and measured by the RKI total score, improved among the mental health professionals and students in both training groups. Differences in training outcomes indicated a better improvement for the PRTP group, independently of the age of the mental health professionals, and as an indirect measure of their level of working experience.

Participants of the PRTP showed a greater improvement in their general attitude and the domains “Roles and Responsibilities” and “Non-linearity of the recovery process” compared to participants of the FPTP, which showed an improvement in the “Role of self-definition and peers in recovery”. Both groups improved in the domain of “Expectations regarding recovery”.

Participants of the PRTP seemed more aware of the peculiarity of this model of care, promoted by the actual scientific literature and widespread knowledge in the Italian mental health service. In the PRTP group, the higher proneness to “take the risk of users’ life choices”, identified by the “Roles and Responsibilities” RKI dimension, could be attributed to our consumer teachers' personal stories. They reported positive experiences, following significant decisions often not approved by the psychiatric staff. The two consumers involved in the PRTP have certainly and mainly contributed talking about their life and illness experiences to the understanding also of the dimension of the “Non-linearity of the recovery process”, highlighting how "ups and downs" can be matched to a recovery process, confirming the value of the “teaching” by a person with lived experience (Byrne et al., 2013).

The improvement in the domain of “Role of self-definition and peers in recovery” of participants of the FPTP compared to participants of the PRTP can be explained by many characteristics of family psychoeducational interventions consistent with the recovery paradigm in mental health, since they are community-based, emphasize achieving personally relevant goals, work on instilling hope and focus on improving natural supports (Glynn et al., 2006). The FPTP stressed the important role of the users as the “main experts” in their family, experts for the experience of their illness. Moreover, carer-based stress management introduced the concept of the “resource group” and its involvement in the treatments. This approach is not limited to the natural family group and can be used with all households or social support groups found in schools, group homes, mental health services or rehabilitation facilities. Close friends can often be more relevant carers than relatives (Casacchia & Roncone, 2014; Falloon et al., 1998), and this perspective is close to the domain of “Role of self-definition and peers in recovery” investigated by the RKI.

In both groups, the RKI domain of “Expectations regarding recovery” increased at the end of the training. These results could be explained by the optimism and renewed motivation that generally follow intense training courses. Also, we hypothesize that the FPTP promoted a wider and effective perspective of successful integrated treatment, that could be further improved by the evolution of the psychoeducational intervention, such as the recovery-oriented salutogenetic approach to foster all the goals that are essential to improve the users’ living conditions (Veltro et al., 2018). Additionally, participants of the PRTP could have developed the awareness that the recovery process follows a “subjective and unique” road for each user, out of the biopsychosocial approach that mental health professionals can provide, where expectations of recovery can become a “faith”, and “a sustained positive expectation” rather than a realistic and “controlled” outcome variable.

The strength of our PRTP could be attributed to users’ participation as teachers and tutors and the format of the program, brief (one only day, eight hours) and easily reproducible at low cost, confirming the findings of Crowe et al. that staff personal recovery orientation can also improve with minimal training and costs (Crowe et al., 2006). Concerning the students of the academic degree in psychiatric rehabilitation techniques, our findings confirmed the increased knowledge of recovery principles and more positive attitudes toward mental illness of medical students shown by Feeney et al. (Feeney et al., 2013) and by the qualitative study of Newton-Howes et al. (2018). Conversely, Gordon et al. (2014) observed that the innovative service-user led tutorials on recovery that they delivered to final year medical students promoted stigmatising attitudes and an extremely pessimistic perspective of users’ outcomes. Indeed, in our study the students of psychiatric rehabilitation techniques in both groups were enthusiastic about their training and showed the same outcome profile as mental health professionals. The choice of such a specific study curriculum positively affected their attitudes towards persons affected by mental illness, without any prejudices.

Additionally, our work shows that it was useful to give an operational frame to participants to support the effort to translate theory into practice, even in services that already adopt good practices. Moreover, staff recovery orientation can improve not only with specific training, as in our PRTP, but also with training like the FPTP, the latter embracing basic personal recovery principles, as the active involvement of users and caregivers, and all the available community resources.

A very recent review identified several education interventions that targeted recovery knowledge and attitudes of mental health professionals’ perceptions regarding recovery-oriented practice (Sreeram et al., 2021). The concept of recovery and recovery-oriented care, crisis prevention and management, the testimony of people with lived experiences, and the effective implementation of the recovery model in mental health settings represent the main educational contents. All studies reported that recovery-related training is important and relevant for enhancing recovery knowledge and attitudes among mental health professionals, regarding recovery-oriented practice (Sreeram et al., 2021). In addition, change in the knowledge and attitudes on recovery seems to improve job satisfaction, personal, and consumer optimism as well, as the development of recovery-related skills (Walsh et al., 2017). However, those changes did seem to have limited influence on service-user and service-level outcomes, (Jackson-Blott et al., 2019; Repique et al., 2016), suggesting the need for ongoing recovery-based intervention to promote recovery-oriented practice in clinical settings.

We could define our intervention that targeted recovery knowledge and attitudes of mental health professionals as the “first-generation” of recovery-oriented care training interventions. Mental health professionals may improve their theoretical model, often already underlining their current practice, but not yet fully conceptualized in such an innovative framework. The “second-generation” of recovery-oriented care training interventions (as the REFOCUS-PULSAR recovery-oriented practice training) is centered on users outcome, indeed a “hard” indicator compared to the “soft” indicator of first-generation studies (Enticott et al., 2021; Meadows et al., 2019). The “second-generation” studies, including longer periods of evaluation and a more ambitious research design, produced encouraging results on users’ recovery promotion.

Our study is a pragmatic and effectiveness study that presents four main methodological limitations. The sample size represents the first limitation, which could also identify the “bias” of selecting in participants more recovery-oriented mental health professionals. Second, our comparison group was represented by participants of a different training course, lacking a proper procedure in selecting our “control” group. Third, we do not know if the staff’s improved “recovery-orientation” will be maintained over time, or, fourth, if it will be translated into their clinical daily practice with significant effect. Despite the conclusions of Sreeram et al. (Sreeram et al., 2021) concerning the limited impact of such recovery-oriented training courses, further studies could improve the format and contents of brief, low-cost recovery-oriented interventions aimed at the effective implementation of the acquired skills in clinical practice.

## Conclusions

Improving the understanding of the paradigm of the personal recovery is an incentive for all mental health professionals to further improve their attitudes in their daily clinical practice towards individuals with mental health disorders and for a recovery-oriented re-organization of mental health services. The personal contribution of the professionals can support such modification, at least in terms of an individual relationship with the user; however, the structural modification of services could take longer. Training the mental health professionals in the principles and values of personal recovery-based practice is considered a key factor in achieving the transformation of mental health services (Bedregal et al., 2006; Crowe et al., 2006; Meehan & Glover, 2009), although we could agree that the “hard” outcome is represented by the users’ appreciation of the recovery-orientation promoted by the trained staff (Bedregal et al., 2006; Crowe et al., 2006; Meadows et al., 2019; Meehan & Glover, 2009).

The positive experience of a recovery structured, short, and low-cost training program can help the dissemination of a new culture for the young mental health professionals students, and refine the cultural frame of more experienced mental health professionals. In a country like Italy, it could be easier than in other countries, considering the existing tradition of community care that needs to be refined concerning the “personal recovery” as an outcome measure. Nevertheless, recovery is difficult “to control” since it is “*defined by the person themself and not other people’s definition of what recovery means*”. And this is the main challenge.
